# A new species of the genus *Leptolalax* (Anura: Megophryidae) from southern Vietnam

**DOI:** 10.24272/j.issn.2095-8137.2018.009

**Published:** 2018-04-28

**Authors:** Tang Van Duong, Dang Trong Do, Chung Dac Ngo, Truong Quang Nguyen, Nikolay A. Poyarkov

**Affiliations:** 1 Vietnam National Museum of Nature, Vietnam Academy of Science and Technology, Hanoi, Vietnam; 2 Department of Vertebrate Zoology, Biological Faculty, Lomonosov Moscow State University, Moscow 119234, Russia; 3 Faculty of Natural Sciences, Phu Yen University, Tuy Hoa, Phu Yen, Vietnam; 4 College of Education, Hue University, Hue, Vietnam; 5 Institute of Ecology and Biological Resources, Vietnam Academy of Science and Technology, Hanoi, Vietnam; 6 Graduate University of Science and Technology, Vietnam Academy of Science and Technology, Cau Giay, Hanoi, Vietnam; 7 Joint Russian-Vietnamese Tropical Research and Technological Center, Nghia Do, Cau Giay, Hanoi, Vietnam

**Keywords:** *Leptolalax macrops***sp. nov.**, Phu Yen Province, Dak Lak Province, Southern coastal region of Vietnam

## Abstract

We describe a new species of megophryid frog from Phu Yen Province in southern Vietnam. *Leptolalax macrops* **sp. nov.** is distinguished from its congeners by a combination of the following morphological attributes: (1) body size medium (SVL 28.0–29.3 mm in three adult males, 30.3 mm in single adult female); (2) supra-axillary glands present, creamy white; ventrolateral glands indistinct; (3) tympanum externally distinct; (4) dorsal skin roughly granular with larger tubercles, dermal ridges on dorsum absent; (5) rudimentary webbing present between fingers I–II and II–III; rudimentary webbing between all toes; fingers and toes without dermal fringes; (6) in life ventral surface greyish-violet with white speckling; (7) supratympanic fold distinct, dark brown in life; (8) iris bicolored, typically golden in upper half, fading to golden green in lower half; (9) tibia short (TbL/SVL 0.44–0.45 in males); and (10) eyes large and protuberant (ED/SVL 0.15–0.16 in males). From all congeners for which comparable sequences are available, the new species differs markedly in the 16S rRNA mitochondrial gene sequence (*P*-distance>5.7%). The new species is currently known only from montane evergreen tropical forests of Song Hinh District, Phu Yen Province, and M’Drak District of Dak Lak Province at elevations of 470–630 m a.s.l.. We suggest the new species should be considered as Data Deficient following the IUCN’s Red List categories. We also report a previously unknown *Leptolalax* mtDNA lineage from an evergreen tropical forest in the Hoa Thinh District of Phu Yen Province, which may also represent an undescribed species.

## INTRODUCTION

Members of the genus *Leptolalax* Dubois, 1983 (Megophryidae Bonaparte, 1850) are widely distributed from northeastern India and southern China southward to the Southeast Asian mainland and Borneo. Knowledge about *Leptolalax* species diversity has strikingly increased in recent decades, from only four in 1983 (Dubois, 1983) to 53 recognized species at present, 31 of which (~60% of total species) have been described in the last 10 years (Frost, 2017). In Vietnam, the number of *Leptolalax* species has increased remarkably from six (Nguyen et al., 2009) to 23 species (Rowley et al., 2016, 2017a, 2017b) within the last decade. However, considering the high morphological similarity of many species within the genus (Rowley et al., 2016) and the poor level of biological exploration of many parts of Indochina, additional taxa likely remain undescribed.

The *L. applebyi* species group is a monophyletic lineage of small to medium-sized frogs (adult SVL<40 mm) inhabiting the southern and central parts of the Annamite (or Truong Son) Mountains in southern Indochina (Poyarkov et al., 2015a; Rowley et al., 2015a, 2016). The group is characterized by morphological similarity of its members and widespread microendemism, with distribution of several lineages restricted to watershed basins (Rowley et al., 2015a). The *L. applebyi* species group currently comprises nine species distributed in the mountains of southern and central Vietnam and adjacent northeastern Cambodia, and include *L. applebyi* Rowley & Cao; *L. ardens* Rowley, Tran, Le, Dau, Peloso, Nguyen, Hoang, Nguyen & Ziegler; *L. bidoupensis* Rowley, Le, Tran, & Hoang; *L. kalonensis* Rowley, Tran, Le, Dau, Peloso, Nguyen, Hoang, Nguyen & Ziegler; *L. maculosus* Rowley, Tran, Le, Dau, Peloso, Nguyen, Hoang, Nguyen & Ziegler; *L. melicus* Rowley, Stuart, Neang & Emmett; *L. pallidus* Rowley, Tran, Le, Dau, Peloso, Nguyen, Hoang, Nguyen & Ziegler; *L. pyrrhops* Poyarkov, Rowley, Gogoleva, Vassilieva, Galoyan, & Orlov; and *L. tadungensis* Rowley, Tran, Le, Dau, Peloso, Nguyen, Hoang, Nguyen & Ziegler (Rowley et al., 2016). Moreover, recent molecular, morphological, and acoustic analyses of the *L. applebyi* species group (Rowley et al., 2015a) revealed another potential new species in the northeastern edges of the Langbian Plateau, indicating that our current understanding of *Leptolalax* diversity in Vietnam is far from complete.

In 2015, during field surveys in the Tay Hoa and Song Hinh districts of the southern area of Phu Yen Province, we encountered two previously unknown populations of *Leptolalax* sp., which also represent the first records of the genus from Phu Yen Province. Morphologically, the newly discovered populations resembled species of the *L. applebyi* group. Consequently, analyses of mtDNA sequences and diagnostic morphological characters suggested that these two populations corresponded to two previously undescribed species of the *L. applebyi* group, one of which is described herein as a new species.

## MATERIALS AND METHODS

### Sample collection

**Figure 1:**
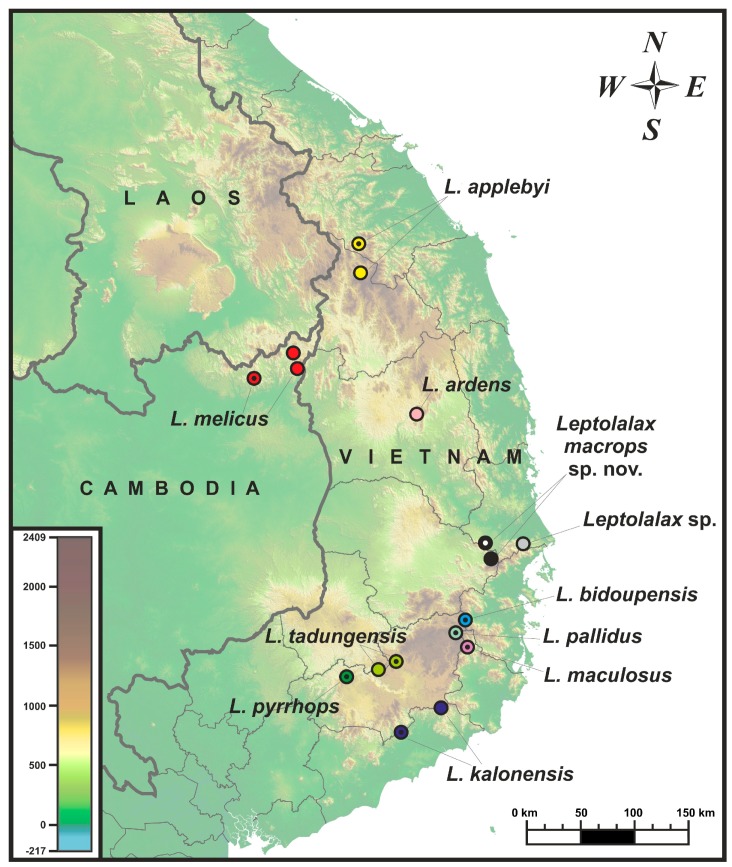
Map of Tay Nguyen Plateau (=Central Highlands) showing distribution of the members of the *Leptolalax applebyi* species group and sampling locations examined in this study

Field surveys were conducted in the forests near Hon Den Mountain, Ea Ly commune, Song Hinh District, and Hoa Thinh commune, Phu Thu Township, Tay Hoa District, in Phu Yen Province, southern Vietnam by Dang Trong Do between July and August 2015 (Figure 1). Specimens were collected by hand from 1900 h to 2300 h. Specimens were photographed in life, then euthanized in a closed vessel with a piece of cotton wool containing ethyl acetate (Simmons, 2002), fixed in 80% ethanol for 5 h, and later transferred to 70% ethanol for permanent storage. Femoral muscle tissue samples were taken prior to preservation for genetic analysis and stored in 96% ethanol. Preserved specimens were deposited in the zoological collection of the Phu Yen University (PYU), Phu Yen Province, Institute of Ecology and Biological Resources (IEBR), Hanoi, Vietnam, and in the herpetological collection of the Zoological Museum of Lomonosov Moscow State University (ZMMU), Moscow, Russia.

**Figure 2:**
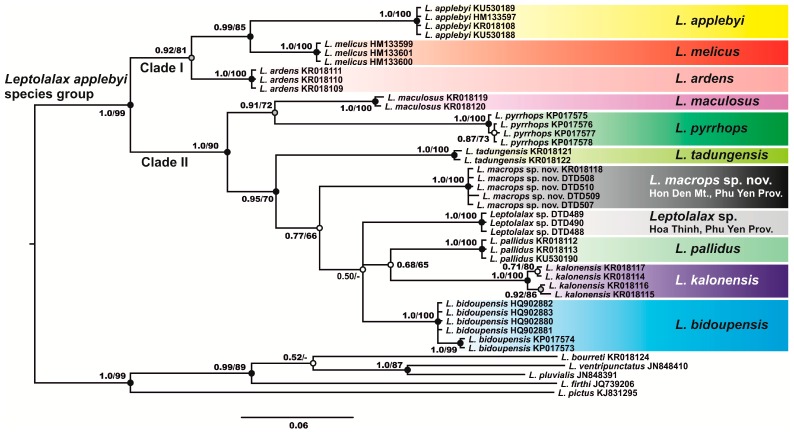
Bayesian inference (BI) phylogram for *Leptolalax applebyi* species group based on analysis of 16S rRNA sequences

Color of locality icons correspond to color bars marking *Leptolalax* species in Figure 2; dot in a center of a circle indicate the type locality of a species.

### Morphological characters

Morphological data were recorded from preserved specimens. Measurements were taken using a digital caliper to the nearest 0.1 mm; the morphometrics of adults and character terminology follow Mahony (2011), Mahony et al. (2013), and Poyarkov et al. (2017). Morphometric abbreviations are: snout to vent length (SVL); head width (HW); head length (HL); eye diameter (ED); tympanum diameter (TYD); eye to tympanum distance (E-T); snout length, measured from snout tip to the anterior corner of the eye (E-S); eye to nare distance (E-N); nare to snout distance (N-S); interorbital distance, measured as the narrowest distance between upper eyelids (IO); internarial distance (IN); upper eyelid width (ELW); forearm length (FAL); hand length (HAL); first finger length (FIL); second finger length (FIIL); third finger length (FIIIL); fourth finger length (FIVL); tibia length (TbL); femur length (FeL); foot length (FOL); tibiotarsal articulation to tip of fourth toe distance (TFOL); and inner metatarsal tubercle length (IMT). Additionally, for description of the type series we measured the distance between anterior orbital borders (IFE); distance between posterior orbital borders (IBE); and length of toes I–V (TI–VL). All measurements were taken on the right side of the examined specimen. Sex was determined by gonadal inspection following dissection.

Statistical analyses were performed with Statistica 6.0 (StatSoft, Inc.). Sexes were separated for subsequent comparisons among samples. One-way analysis of variance (ANOVA) and Duncan’s post-hoc tests were used for morphometric comparisons. A significance level of 95% was used of all statistical tests.

Comparative morphological data were obtained from museum specimens of *Leptolalax* and (when available) photographs of these specimens in life (Appendix I). Data on the morphological characters of *Leptolalax* species are also available from the following literature: *L. aereus* Rowley, Stuart, Richards, Phimmachak & Sivongxay (Rowley et al., 2010b); *L. alpinus* Fei, Ye & Li (Fei et al., 1990, 2009, 2010), *L. applebyi* (Rowley & Cao, 2009; Rowley et al., 2016); *L. arayai* Matsui (Matsui, 1997), *L. ardens* (Rowley et al., 2016); *L. bidoupensis* (Rowley et al., 2011, 2016); *L. botsfordi* Rowley, Dau & Nguyen (Rowley et al., 2013); *L. bourreti* Dubois (Dubois, 1983; Ohler et al., 2011); *L. croceus* Rowley, Hoang, Le, Dau & Cao (Rowley et al., 2010c); *L. dringi* Dubois (Dubois, 1987; Inger et al., 1995); *L. eos* Ohler, Wollenberg, Grosjean, Hendrix, Vences, Ziegler & Dubois (Ohler et al., 2011); *L. firthi* Rowley, Hoang, Dau, Le & Cao (Rowley et al., 2012); *L. fritinniens* Dehling & Matsui (Dehling & Matsui, 2013) *L. fuliginosus* Matsui (Matsui, 2006); *L. gracilis* (Günther) (Günther, 1872; Inger & Stuebing, 2005); *L. hamidi* Matsui (Matsui, 1997); *L. heteropus* (Boulenger) (Boulenger, 1900); *L. isos* Rowley, Stuart, Neang, Hoang, Dau, Nguyen & Emmett (Rowley et al., 2015b); *L. kajangensis* Grismer, Grismer & Youmans (Grismer et al., 2004); *L. kalonensis* (Rowley et al., 2016); *L. kecil* Matsui, Belabut, Ahmad & Yong (Matsui et al., 2009); *L. khasiorum* Das, Tron, Rangad & Hooroo (Das et al., 2010); *L. lateralis* (Anderson) (Anderson, 1871; Humtsoe et al., 2008), *L. laui* Sung, Yang & Wang (Sung et al., 2014), *L. liui* Fei & Ye (Fei et al., 1990, 2009, 2010); *L. maculosus* (Rowley et al., 2016); *L. maoershanensis* Yuan, Sun, Chen, Rowley & Che (Yuan et al., 2017); *L. marmoratus* Matsui, Zainudin & Nishikawa (Matsui et al., 2014b); *L. maurus* Inger, Lakim, Biun & Yambun (Inger et al., 1997); *L. melanoleucus* Matsui (Matsui, 2006); *L. melicus* (Rowley et al., 2010a, 2016); *L. minimus* (Taylor) (Taylor, 1962; Ohler et al., 2011); *L. nahangensis* Lathrop, Murphy, Orlov & Ho (Lathrop et al., 1998); *L. nokrekensis* (Mathew & Sen) (Mathew & Sen, 2009); *L. nyx* Ohler, Wollenberg, Grosjean, Hendrix, Vences, Ziegler & Dubois (Ohler et al., 2011); *L. oshanensis* (Liu) (Fei et al., 2009, 2010; Liu, 1950); *L. pallidus* (Rowley et al., 2016); *L. pelodytoides* Boulenger (Boulenger, 1893, 1908; Ohler et al., 2011); *L. petrops* Rowley, Dau, Hoang, Le, Cutajar & Nguyen (Rowley et al., 2017a); *L. pictus* Malkmus (Malkmus, 1992; Malkmus et al., 2002); *L. platycephalus* Dehling (Dehling, 2012); *L. pluvialis* Ohler, Marquis, Swan & Grosjean (Ohler et al., 2000, 2011), *L. puhoatensis* Rowley, Dau & Cao (Rowley et al., 2017b); *L. pyrrhops* (Poyarkov et al., 2015a); *L. sabahmontanus* Matsui, Nishikawa & Yambun (Matsui et al., 2014a), *L. solus* Matsui (Matsui, 2006); *L. sungi* Lathrop, Murphy, Orlov & Ho (Lathrop et al., 1998); *L. tadungensis* (Rowley et al., 2016); *L. tamdil* Sengupta, Sailo, Lalremsanga, Das & Das (Sengupta et al., 2010); *L. tengchongensis* Yang, Wang, Chen & Rao (Yang et al., 2016); *L. tuberosus* Inger, Orlov & Darevsky (Inger et al., 1999; Rowley et al., 2010c); *L. ventripunctatus* Fei, Ye & Li (Fei et al., 1990, 2009, 2010; Ohler et al., 2011); and *L. zhangyapingi* Jiang, Yan, Suwannapoom, Chomdej & Che (Jiang et al., 2013). However, due to the considerable undiagnosed diversity within the family Megophryidae (Chen et al., 2017; Mahony et al., 2017; Rowley et al., 2015a, 2016), we relied on examination of topotypic material and/or original species descriptions.

### DNA isolation, PCR, and sequencing

Total DNA was extracted from muscle tissue using standard phenol-chloroform extraction (Hillis et al., 1996), followed by isopropanol precipitation. We amplified a 454–474-bp length fragment of the 16S rRNA mitochondrial gene, which has been successfully applied for DNA-identification of cryptic diversity within the genus *Leptolalax* (Poyarkov et al., 2015a; Rowley et al., 2015a, 2016, 2017a, 2017b). The 16S rRNA-gene fragment was amplified using ScreenMix-HS (Evrogen, Russia) following the manufacturer’s instructions. The PCR contained 6 μL of ScreenMix-HS, 21 μL of water, 0.9 μL of each primer at a concentration of 10 pmol/μL, and 1.2 μL of template DNA at a concentration up to ca. 100 ng DNA/μL in a 30 μL reaction volume.

The primers used for PCR and sequencing were: 16SL-1 (5${}^{\prime }$-CTGACCGTGCAAAGGTAGCGTAATCACT-3${}^{\prime }$; forward) and 16SH-1 (5${}^{\prime }$-CTCCGGTCTGAACTCAGATCACGTAGG-3${}^{\prime }$; reverse) (Hedges, 1994). The PCR conditions followed Poyarkov et al. (2015b). The amplification protocols included: 94 ${}^{\circ }$C for 5 min of initial denaturation; followed with 35 cycles of denaturation at 94 ${}^{\circ }$C for 1 min, annealing at 55 ${}^{\circ }$C for 1 min, and extension at 72 ${}^{\circ }$C for 1 min; and a final extension at 72 ${}^{\circ }$C for 10 min. The obtained PCR products were loaded onto 1% agarose gels and visualized in the presence of ethidium bromide in a Dark Reader Transilluminator (Clare Chemical, USA). If distinct bands were produced, they were sent to Evrogen (Moscow, Russia) for subsequent purification and sequencing in both directions. The obtained sequences were checked by eye using chromatogram editor software DNA Baser v4.20.0; primer sequences were removed, and the edited sequences were submitted to GenBank under the accession numbers MG787987–MG787993 (Table 1).

**Table 1:** Specimens, localities, museum voucher IDs, and GenBank accession Nos. of the *Leptolalax applebyi* group members and *Leptolalax* species outgroup used for molecular analyses

Species	Locality	Voucher No.	GenBank accession No.
*Leptolalax applebyi*	Song Thanh, Quang Nam Prov., Vietnam	AMS R171703	HM133597
*L. applebyi*	Ngoc Linh Mt., Kon Tum Prov., Vietnam	AMS R173778	KR018108
*L. applebyi*	Ngoc Linh Mt., Kon Tum Prov., Vietnam	AMS R173737	KU530188
*L. applebyi*	Ngoc Linh Mt., Kon Tum Prov., Vietnam	AMS R173735	KU530189
*L. ardens*	Kon Ka Kinh, Gia Lai Prov., Vietnam	AMS R176454	KR018109
*L. ardens*	Kon Ka Kinh, Gia Lai Prov., Vietnam	AMS R176463	KR018110
*L. ardens*	Kon Ka Kinh, Gia Lai Prov., Vietnam	AMS R176467	KR018111
*L. bidoupensis*	Hon Giao Mt., Lam Dong Prov., Vietnam	ASM R173133	HQ902880
*L. bidoupensis*	Hon Giao Mt., Lam Dong Prov., Vietnam	AMS R173134	HQ902881
*L. bidoupensis*	Hon Giao Mt., Lam Dong Prov., Vietnam	NCSM77320	HQ902882
*L. bidoupensis*	Hon Giao Mt., Lam Dong Prov., Vietnam	NCSM77321	HQ902883
*L. bidoupensis*	Bidoup Mt., Lam Dong Prov., Vietnam	ZMMU NAP- 01453	KP017573
*L. bidoupensis*	Bidoup Mt., Lam Dong Prov., Vietnam	ZMMU NAP-01458	KP017574
*L. kalonensis*	Song Luy, Binh Thuan Prov., Vietnam	IEBRA2014.15	KR018114
*L. kalonensis*	Song Luy, Binh Thuan Prov., Vietnam	AMNHA191762	KR018115
*L. kalonensis*	Song Luy, Binh Thuan Prov., Vietnam	IEBRA2014.16	KR018116
*L. kalonensis*	Song Luy, Binh Thuan Prov., Vietnam	AMNHA191765	KR018117
*L. macrops* **sp. nov.**	Dak Lak Prov., Vietnam	AMS R177663	KR018118
*L. macrops* **sp. nov.**	Hon Den Mt., Phu Yen Prov., Vietnam	IEBR A.2017.9	MG787990
*L. macrops* **sp. nov.**	Hon Den Mt., Phu Yen Prov., Vietnam	PYU DTD-508	MG787991
*L. macrops* **sp. nov.**	Hon Den Mt., Phu Yen Prov., Vietnam	PYU DTD-509	MG787992
*L. macrops* **sp. nov.**	Hon Den Mt., Phu Yen Prov., Vietnam	ZMMU A-5823	MG787993
*L. maculosus*	Phuoc Binh, Ninh Thuan Prov., Vietnam	AMS R177660	KR018119
*L. maculosus*	Phuoc Binh, Ninh Thuan Prov., Vietnam	ZFMK 96600	KR018120
*L. melicus*	Virachey, Ratanakiri Prov., Cambodia	MVZ 258197	HM133599
*L. melicus*	Virachey, Ratanakiri Prov., Cambodia	MVZ 258198	HM133600
*L. melicus*	Virachey, Ratanakiri Prov., Cambodia	MVZ 258199	HM133601
*L. pallidus*	Gia Rich, Lam Dong Prov., Vietnam	USN00510	KR018112
*L. pallidus*	Gia Rich, Lam Dong Prov., Vietnam	USN00512	KR018113
*L. pallidus*	Gia Rich, Lam Dong Prov., Vietnam	USN00511	KU530190
*L. pyrrhops*	Loc Bac, Lam Dong Prov., Vietnam	ZMMU A-5208	KP017575
*L. pyrrhops*	Loc Bac, Lam Dong Prov., Vietnam	ZMMU A-4873-1	KP017576
*L. pyrrhops*	Loc Bac, Lam Dong Prov., Vietnam	ZMMU A-4873-2	KP017577
*L. pyrrhops*	Loc Bac, Lam Dong Prov., Vietnam	ZMMU A-4873-3	KP017578
*L. tadungensis*	Ta Dung, Dak Nong Prov., Vietnam	USN00515	KR018121
*L. tadungensis*	Ta Dung, Dak Nong Prov., Vietnam	USN00517	KR018122
*Leptolalax* sp.	Hoa Thinh, Phu Yen Prov., Vietnam	PYU DTD-488	MG787987
*Leptolalax* sp.	Hoa Thinh, Phu Yen Prov., Vietnam	ZMMU A-5824	MG787988
*Leptolalax* sp.	Hoa Thinh, Phu Yen Prov., Vietnam	PYU DTD-490	MG787989
*L. bourreti*	Lao Cai Prov., Vietnam	AMS R177673	KR018124
*L. firthi*	Kon Tum Prov., Vietnam	AMS R 176524	JQ739206
*L. pictus*	Malaysia, Borneo	UNIMAS 8705	KJ831295
*L. pluvialis*	Lao Cai Prov., Vietnam	MNHN1999.5675	JN848391
*L. ventripunctatus*	Phongsaly Prov., Laos	MNHN 2005.0116	JN848410

### Phylogenetic analyses

For phylogenetic analyses of the *L. applebyi* species group, we used 32 published sequences of 16S rRNA (Poyarkov et al., 2015a; Rowley et al., 2015a, 2016) and seven newly obtained sequences of *Leptolalax* sp. from Phu Yen Province (Table 1). In total, a dataset of 39 ingroup sequences was used for the analyses. Sequences of *L. ventripunctatus*, *L. bourreti*, *L. pluvialis*, *L. firthi*, and *L. pictus*, representing different species groups within *Leptolalax*, were used as outgroup taxa following Rowley et al. (2016).

Sequences of 44 specimens of *Leptolalax* representatives, with a total length of up to 1 046 bp, were included in the final alignment and subjected to phylogenetic analyses. Sequences were initially aligned using ClustalW (Thompson et al., 1997) in Bioedit 7.0.5 (Hall, 1999) with default parameters. Mean uncorrected genetic distances (*P*-distances) between sequences and species were calculated using MEGA 7.0 (Kumar et al., 2016). PartitionFinder v.1.1.0 (Lanfear et al., 2012) was applied to estimate the optimal evolutionary models used for dataset analysis. The best-fitting model was the GTR+I+G model of DNA evolution, as suggested by the Akaike Information Criterion (AIC), corrected Akaike Information Criterion (AICc), and Bayesian Information Criterion (BIC).

The matrilineal genealogy was inferred using Bayesian inference (BI) and maximum likelihood (ML) algorithms. The BI analyses were conducted in MrBayes v.3.1.2 (Huelsenbeck & Ronquist, 2001; Ronquist & Huelsenbeck, 2003); Metropolis-coupled Markov chain Monte Carlo (MCMCMC) analyses were run with one cold chain and three heated chains for ten million generations and sampled every 1 000 generations. Five independent MCMCMC runs were performed and 1 000 trees were discarded as burn-in. Confidence in topology was assessed by posterior probability (BI PP, Huelsenbeck & Ronquist, 2001). The ML analyses were conducted using Treefinder (Jobb et al., 2004) and confidence in node topology was tested by non-parametric bootstrapping with 1 000 replicates (ML BS, Felsenstein, 1985). We *a priori* regarded tree nodes with bootstrap (ML BS) values of 70% or greater and Bayesian posterior probabilities (BI PP) values over 0.95 as sufficiently resolved (Felsenstein, 2004; Hillis & Bull, 1993; Huelsenbeck & Hillis, 1993). The ML BS values between 70% and 50% (BI PP between 0.95 and 0.90) were treated as tendencies and nodes with ML BS values below 50% (BI PP below 0.90) were regarded as unresolved.

## RESULTS

### Sequence variation

The 16S rRNA dataset contained 39 ingroup and five outgroup *Leptolalax* sequences. The final alignment consisted of 1 075 sites, with 617 conserved sites and 413 variable sites, 139 of which were parsimony-informative; the transition-transversion bias (R) was estimated as 2.14 (all data given for ingroup only). Substitution rates were estimated under the General Time Reversible (GTR) model (+I+G). Nucleotide frequencies were A=31.23%, T=24.47%, C=24.27%, and G=20.03%.

### Phylogenetic relationships

Phylogenetic analysis results of the 16S rRNA gene fragment are shown in Figure 2. The ML and BI phylogenetic analyses showed essentially similar topologies, which only differed slightly in associations at poorly supported basal nodes.

Values at nodes correspond to BI PP / ML BS support values, respectively. Sequences of *L. pictus*, *L. pluvialis*, *L. ventripunctatus*, *L. firthi*, and *L. bourreti* were used as outgroups.

In general, the topology of the BI cladogram was consistent with results reported in previous work (Poyarkov et al., 2015a; Rowley et al., 2015a, 2016), suggesting monophyly of the *L. applebyi* species group (node support values 1.0/99, hereafter given for BI PP/ML BS, respectively) and the presence of two major lineages within it. Clade I encompassed three species inhabiting the Tay Nguyen (Kon Tum) Plateau in central Vietnam and northeastern Cambodia: namely, *L. applebyi*, *L. ardens*, and *L. melicus* (Figure 2). Clade II comprised the remaining *L. applebyi* group species from the Langbian (Da Lat) Plateau of the Southern Annamite Mountains. Phylogenetic relationships within Clade II were not sufficiently resolved: there was a tendency toward a more distant position for *L. pyrrhops* and *L. maculosus*, with the remaining lineages forming a monophyletic group (0.95/70). The two newly discovered populations of *Leptolalax* sp. from Phu Yen Province formed two independent mtDNA matrilines: that is, the Hon Den Mt. lineage and Hoa Thinh lineage. The sequence of *Leptolalax* sp. from Dak Lak Province (indicated as “molecular lineage 7” in Rowley et al., 2015a) shared the same mtDNA haplotype as the *Leptolalax* sp. population from Hon Den Mt., Phu Yen Province, suggesting that these two populations are conspecific.

### Genetic distances

The uncorrected *P*-distances among and within the 16S rRNA gene fragment sequences of the studied *Leptolalax* species are shown in Table 2. The observed interspecific distances within the *L. applebyi* group members ranged from 4.4% (between *L. kalonensis* and *L. pallidus*) to 10.3% (between *L. applebyi* and *L. pyrrhops*) of substitutions. The uncorrected genetic *P*-distances in the ingroup and outgroup comparisons partly overlapped: genetic distances between the *L. applebyi* group members versus the *Leptolalax* taxa outgroup ranged from 9.2% (between *L. maculosus* and *L. pluvialis*) to 15.4% (between *L. kalonensis* and *L. firthi*).

**Table 2:** Uncorrected *P*-distances (percentages) between the examined 16S rRNA sequences of the *Leptolalax applebyi* group members (1–11) and *Leptolalax* species outgroup (12–13)

	Species	1	2	3	4	5	6	7	8	9	10	11	12	13	14	15	16
**1**	*Leptolalax macrops* sp. nov.	**0.1**															
**2**	*L. ardens*	**6.4**	**0.0**														
**3**	*L. bidoupensis*	**5.7**	7.3	**0.0**													
**4**	*L. kalonensis*	**6.7**	8.7	4.9	**0.4**												
**5**	*L. maculosus*	**6.3**	5.9	6.3	6.6	**0.2**											
**6**	*L. melicus*	**8.2**	4.2	6.9	9.2	7.7	**0.0**										
**7**	*L. pallidus*	**5.8**	6.9	4.5	4.4	6.3	8.0	**0.0**									
**8**	*L. pyrrhops*	**7.2**	7.4	7.7	6.9	5.5	10.2	6.3	**0.2**								
**9**	*L. tadungensis*	**6.5**	7.0	5.3	5.3	6.0	7.9	5.5	6.0	**0.2**							
**10**	*L. applebyi*	**8.7**	5.3	6.9	9.4	8.2	4.9	7.6	10.3	8.0	**0.0**						
**11**	*Leptolalax* sp.	**5.7**	8.3	4.5	5.8	7.3	8.3	4.5	7.4	5.9	8.5	**0.0**					
**12**	*L. bourreti*	**12.5**	10.4	12.2	13.3	11.1	10.4	12.4	13.3	12.9	10.9	13.6	—				
**13**	*L. pluvialis*	**10.8**	10.4	10.7	11.3	9.2	10.0	10.8	11.1	9.8	10.0	11.0	7.6	—			
**14**	*L. pictus*	**12.1**	11.3	13.0	12.9	12.6	12.0	12.0	13.7	12.8	11.8	14.3	14.0	12.3	—		
**15**	*L. ventripunctatus*	**12.5**	10.5	12.8	13.2	10.3	11.2	12.4	11.2	11.7	11.4	13.2	8.5	5.4	11.5	—	
**16**	*L. firthi*	**13.5**	12.5	13.4	15.4	12.6	12.7	13.7	13.7	12.7	13.0	14.2	10.4	10.3	12.5	8.8	—

Mean uncorrected intraspecific *P*-distances of the ingroup are shown on the diagonal.

The newly discovered population of *Leptolalax* sp. from Hon Den Mt., Song Hinh District, was clearly distinct from all other group members in the examined 16S rRNA fragment sequences and appeared to be most closely related to *L. bidoupensis* from the eastern edges of Langbian Plateau (Lam Dong and Khanh Hoa provinces) and to a *Leptolalax* sp. population from Hoa Thinh, Tay Hoa District (Phu Yen Province) (*P*-distance=5.7% for both comparisons). The *Leptolalax* sp. population from Hoa Thinh was genetically closer to *L. bidoupensis* and *L. pallidus*, with a *P*-distance value of 4.5% (both species from eastern Langbian Plateau).

The observed pairwise divergence in 16S rRNA was greater than that usually seen among species of anurans (Vences et al., 2005a, 2005b; Vieites et al., 2009) and was higher than distances between some other recognized species of the 1*L. applebyi* group (e.g., 4.4% between *L. pallidus* and *L. kalonensis* and 4.2% between *L. ardens* and *L. melicus*) (Table 2).

Intraspecific genetic *P*-distances were 0.0% in the *Leptolalax* sp. population from Hoa Thinh, and 0.1% in the *Leptolalax* sp. from Hon Den Mt.; the five examined specimens of the latter species of *Leptolalax* from Dak Lak and Phu Yen provinces had two haplotypes of the 16S rRNA gene fragment.

### Taxonomy

Our molecular data clearly indicated that the two recently discovered populations of *Leptolalax* sp. from Song Hinh (Hon Den Mt.) and Tay Hoa (Hoa Thinh) districts of Phu Yen Province belong to two independent mtDNA lineages, clearly distinct from each other and from the remaining members of the *L. applebyi* species group. Despite geographical proximity (~30 km between Hon Den Mt. and Hoa Thinh), these two localities cradle distinct species of *Leptolalax*, and both appear to be new to science. These two potentially new species were assigned to the Langbian Plateau clade of the *L. applebyi* species group and appear to be closely related to *L. pallidus*, *L. kalonensis*, and *L. bidoupensis*. At the same time, the population of Hon Den Mt. appears to be conspecific to a *Leptolalax* sp. found in the eastern part of Dak Lak Province (~30 km between localities).

Lacking enough material for morphological comparisons, we tentatively indicate the *Leptolalax* sp. population of Hoa Thinh (Tay Hoa District) as a candidate new species *sensu* Vieites et al. (2009); further morphological and acoustic studies are necessary to clarify its taxonomic status. Based on genetic differentiation, phylogenetic analyses of a 16S rRNA fragment of mtDNA, and analyses of diagnostic morphological characters (see below in “Comparisons”), the population of *Leptolalax* from Hon Den Mt. in Phu Yen Province of southern Vietnam clearly represents a new species, which we describe as follows.

***Leptolalax macrops* sp. nov.**

**Figure 3:**
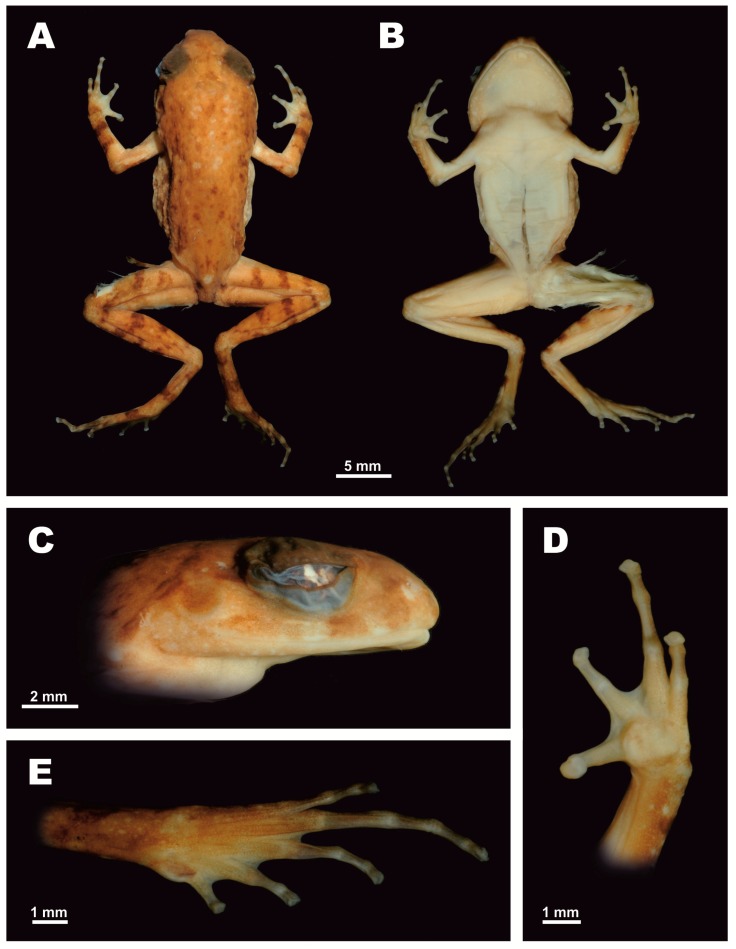
Male holotype of *Leptolalax macrops* sp. nov. (PYU DTD-508) in preservative (Photos by Nikolay A. Poyarkov)

**Figure 4:**
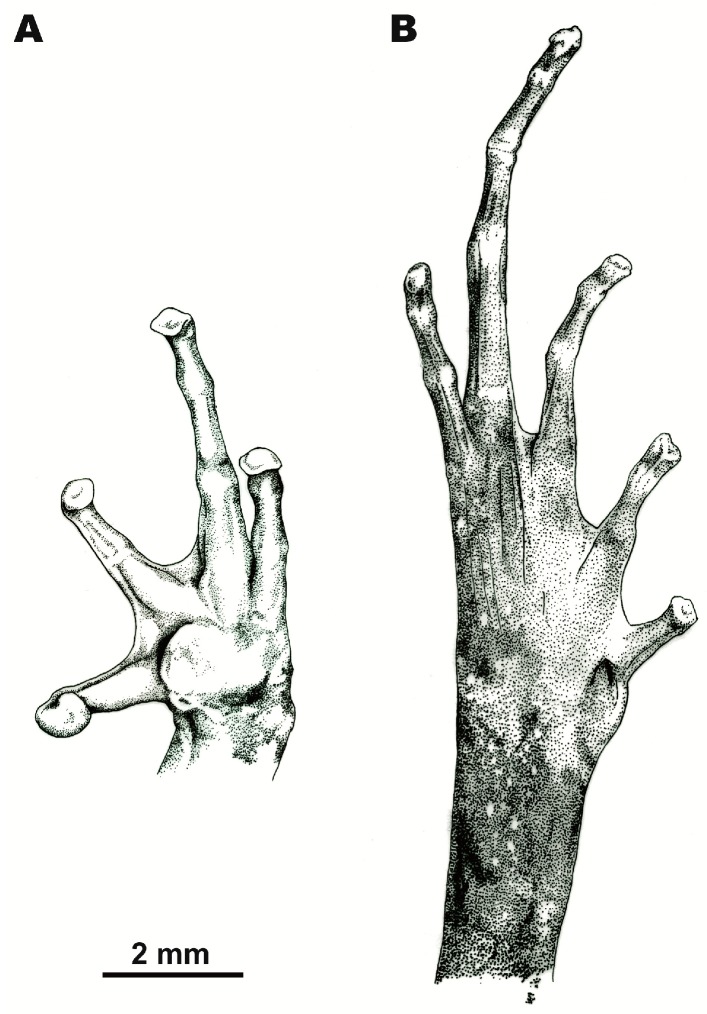
Volar surface of left hand and plantar surface of right foot of preserved holotype of *Leptolalax macrops* sp. nov. (PYU DTD-508) (Drawings by Valentina D. Kretova)

**Figure 5:**
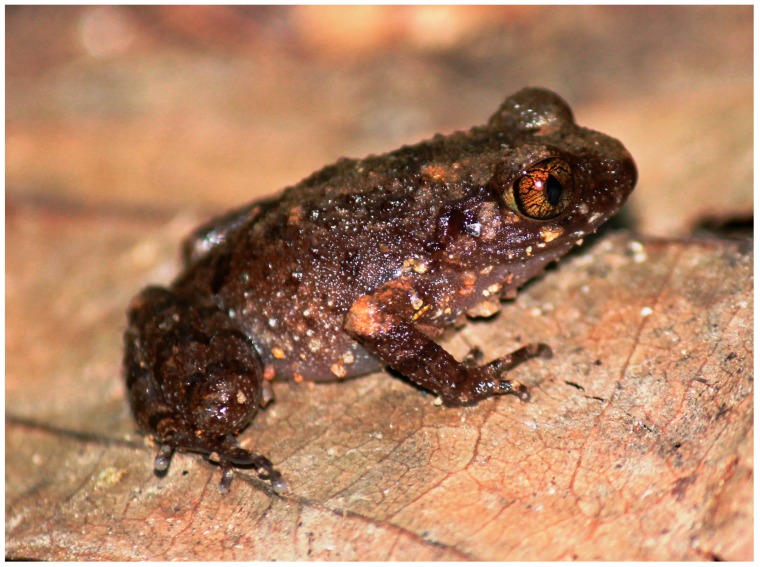
Male paratype of *Leptolalax macrops* sp. nov. (IEBR A.2017.9) in life (Photo taken *in situ*. Photo by Dang Trong Do)

Figure 3, Figure 4 and Figure 5, Table 3.

A: Dorsal view of body; B: Ventral view of body; C: Lateral view of head; D: Volar view of left hand; E: Plantar view of right foot.

Scale bar: 2 mm.

**Table 3:** Measurements of the type series of *Leptolalax macrops* sp. nov.

Specimen character	Holotype PYU DTD-508	Paratype IEBR A.2017.9	Paratype ZMMU A-5823	Mean(M)	*SD* (M)	Paratype PYU DTD-509
Sex	M	M	M			F
SVL	28.0	29.3	28.3	28.6	*0.7*	30.3
HW	9.9	10.6	10.1	10.2	*0.4*	11.2
HL	10.7	10.9	10.7	10.8	*0.1*	11.9
ED	4.3	4.5	4.5	4.4	*0.1*	4.8
TyD	1.9	1.9	1.9	1.9	*0.0*	2.1
E-T	0.8	0.8	0.8	0.8	*0.0*	1.0
E-S	3.7	4.0	4.1	3.9	*0.2*	4.3
E-N	2.0	2.0	2.1	2.0	*0.1*	2.4
N-S	1.4	1.4	1.5	1.5	*0.1*	1.9
IO	2.9	3.0	2.9	2.9	*0.1*	3.2
IN	2.3	2.3	2.4	2.3	*0.0*	2.5
ELW	2.7	2.9	2.8	2.8	*0.1*	3.1
FAL	7.1	7.7	7.3	7.3	*0.3*	7.9
HAL	6.2	6.9	6.4	6.5	*0.4*	7.1
FIL	1.8	1.9	1.9	1.9	*0.1*	2.0
FIIL	2.6	2.8	2.5	2.6	*0.2*	2.9
FIIIL	4.5	4.8	4.6	4.6	*0.2*	5.2
FIVL	1.8	2.3	2.3	2.2	*0.3*	3.0
TbL	12.5	13.3	12.8	12.8	*0.4*	14.3
FeL	11.9	13.1	12.6	12.5	*0.6*	13.8
FOL	11.2	12.5	11.5	11.7	*0.7*	13.9
TFOL	17.0	19.4	17.5	18.0	*1.3*	20.3
IMT	1.8	2.2	2.1	2.0	*0.2*	2.4
SVL/HL	2.6	2.7	2.6	2.6	*0.0*	2.5
HL/HW	1.1	1.0	1.1	1.1	*0.0*	1.1
SVL/TbL	2.2	2.2	2.2	2.2	*0.0*	2.1
ED/SVL	0.2	0.2	0.2	0.2	*0.0*	0.2

*SD*: standard deviation; M: male; F: female; for other abbreviations see the Materials and Methods. All measurements are in mm.

**Chresonymy:** *Leptolalax* sp. [molecular lineage 7] — Rowley et al., 2015a: 10, 12.

**Holotype:** PYU DTD-508 (field no. DTD-0508), adult male collected from Suoi Khi Stream, Hon Den Mt., Ea Ly and Ea Trol Commune border, Song Hinh District, Phu Yen Province, Vietnam (N12${}^{\circ }$52${}^{\prime }$47.0${}^{\prime \prime }$, E108${}^{\circ }$49${}^{\prime }$51.1${}^{\prime \prime }$; at an elevation of 500 m a.s.l.), collected by Dang Trong Do on 18 August 2015 at 2100 h.

**Paratypes:** IEBR A.2017.9 (field no. DTD-507) and ZMMU A-5823 (field no. DTD-510), two adult males, and PYU DTD-509 (field no. DTD-509), one adult female, collected from the same locality as the holotype at elevations between 471 and 630 m a.s.l. by Dang Trong Do on 18 August 2015 from 1900 h to 2300 h.

**Diagnosis:** The species is assigned to the genus *Leptolalax* based on the following characters: (1) finger tips rounded; (2) elevated inner metacarpal tubercle present, not continuous onto thumb; (3) body with macroglands (including supra-axillary, pectoral, and femoral glands); (4) vomerine teeth absent; (5) tubercles on eyelids present; and (6) anterior tip of snout with whitish vertical bar (Delorme et al., 2006; Dubois, 1980, 1983; Lathrop et al., 1998; Matsui, 1997, 2006; Rowley et al., 2013). *Leptolalax macrops* **sp. nov.** is distinguished from its congeners by a combination of the following morphological characters: (1) body size medium (SVL 28.0–29.3 mm in three adult males, 30.3 mm in single adult female); (2) supra-axillary glands present, creamy white; ventrolateral glands indistinct; (3) tympanum externally distinct; (4) dorsal skin roughly granular with larger tubercles, dermal ridges on dorsum absent; (5) rudimentary webbing present between fingers I–II and II–III; rudimentary webbing between all toes; fingers and toes without dermal fringes; (6) in life ventral surface greyish-violet with rare white speckling; (7) supratympanic fold dark brown; (8) iris bicolored, typically golden in upper half, fading to golden green in lower half; (9) tibia short (TbL/SVL 0.44–0.45); and (10) eyes large and protuberant (ED/SVL 0.15–0.16). The new species is also markedly distinct from all congeners for which comparable 16S rRNA mitochondrial gene sequences are available (uncorrected genetic *P*-distance>5.7%).

**Etymology:** Specific epithet “*macrops*” is a noun in the nominative case, derived from Greek “*macros*” for “large” and “*ops*” for “eye”, in reference to its comparatively large eye size.

**Recommended vernacular names:** We recommend “Big-eyed Litter Frog” as the common English name of the new species and the common name in Vietnamese as “*Cóc mày mắt to*”.

**Description of holotype:** Medium-sized *Leptolalax* specimen (SVL 28.0 mm); body and head in good state of preservation, fingers and toes partially dehydrated due to ethanol preservation (Figure 3A,B). Left thigh of holotype damaged, skin on ventral surface of thigh dissected, with a significant portion of femoral muscle removed, dissection length ca. 10 mm. Belly also dissected medially, dissection length ca. 9 mm, testes can be seen through dissection.

**Head:** Head flattened, slightly longer than wide (HW/HL 92.7%), top of head weakly concave; snout short (E-S/HL 34.2%), slightly projecting beyond margin of lower jaw; slightly truncated in dorsal view (Figure 3A), obtusely rounded in ventral view (Figure 3B), gently sloping and rounded in profile (Figure 3C); nostril ovoid, oblique, slightly closer to tip of snout than to eye (Figure 3C; N-S/E-N 72.1%); canthus rostralis distinct, bluntly rounded; loreal region slightly concave; eyes very large (ED/HL 40.3%), eye diameter greater than snout length (ED/E-S 117.8%), notably protuberant in dorsal view in life (eyeballs depressed down in preserved holotype, Figure 3C); pupil vertical, diamond-shaped; tympanum distinct, round with vertical diameter equal to horizontal diameter; tympanum small, less than half eye diameter (TyD/ED 44.0%); tympanic rim indistinct, not elevated relative to skin of temporal region; pineal ocellus absent; vomerine teeth absent; vocal sac gular, vocal sac openings small, oval, and slit-like, located laterally in corners of mouth floor; tongue long, wide, with free posterior end, heart-shaped with shallow medial notch at posterior tip; supratympanic fold well-developed forming distinct glandular ridge, running from posterior corner of eye posteriorly toward dorsal edge of tympanum, gently curving ventrally toward axilla, bearing several flat tubercles (Figure 3C).

**Forelimbs:** Forelimbs thin, slender; finger tips in life rounded, but appear slightly enlarged and truncate in preservative due to partial dehydration, finger tips approximately same width as distal finger articulation; relative finger lengths: IV=I<II<III; nuptial pad indistinct; subarticular tubercles absent, replaced with low dermal ridges prominent on fingers II–IV; inner metacarpal tubercle large, fused with outer one, forming single bulging callous structure, prominent on palmar surface (maximal length 1.3 mm); border between inner and outer metacarpal tubercles indistinct; fingers in life lack dermal fringing, basal webbing present between fingers I and II and fingers II and III, absent between fingers III and IV (Figure 3D, Figure 4A).

**Hindlimbs:** Hindlimbs slender, short, tibia less than half snout-vent length (TbL/SVL 44.5%); tibiotarsal articulation of adpressed limb reaching eye-level; toe tips round in life, slightly truncate in ethanol preservative due to partial dehydration; relative toe lengths: I<V<II<III<IV; subarticular tubercles absent, replaced by dermal ridges, distinct on all toes and continuing to metatarsus of toes III–V; inner metatarsal tubercle large, oval-shaped, nearly two times longer than wide (IMT to width of inner metatarsal tubercle ratio 186.4%), outer metatarsal tubercle absent; toes without lateral dermal fringes; basal webbing present between all five toes, webbing well-developed between toes I and II, II and III, and III and IV (reaching level of proximal finger articulation), and somewhat reduced between toes IV and V (Figure 3E, Figure 4B).

**Skin texture and skin glands:** Skin on entire dorsum roughly granular, covered in tubercles of varying sizes, smaller dorsolaterally; upper eyelids with numerous small rounded tubercles (flattened in preservative, Figure 3C), snout smooth; ventral skin smooth; pectoral gland distinct in preservative and in life, round, located near axilla, 0.9 mm in diameter (Figure 3B); femoral gland oval, small, 0.7 mm in diameter, located on posteroventral surface of thigh, approximately five times closer to knee than to vent; supra-axillary gland present, protuberant, creamy white, located in axillary region dorsally from insertion of forelimb, 0.9 mm in diameter; ventrolateral glands indistinct.

**Coloration in life:** Dorsal surfaces of head and trunk dark brownish-grey with indistinct dark brown blotches scattered on posterior part of dorsum and between eyes; interorbital region with dark bar with indistinct edges; several light brown blotches of irregular shape and indistinct edges on anterior part of upper eyelids, scapular region, and sacrum. Dorsal surfaces of forelimbs and hindlimbs brownish-grey, elbows and upper arms dorsally much lighter with coppery orange background. Dark brown line running along canthus rostralis through eye, and continuing below supratympanic fold, terminating above axilla, encompassing nare, loreal region but not tympanum; tympanum lighter than surrounding skin of temporal region. Faint transverse dark brown bars on dorsal surface of thighs, tibia, tarsus, lower arms, fingers, and toes. Small indistinct dark brown blotches on flanks. Tiny whitish flecks scattered on dorsolateral sides of body from groin to axilla. Belly and chest greyish-violet with rare white speckling on entire ventral surface, including throat, arms, and legs. Supra-axillary gland creamy white; femoral glands whitish; pectoral glands white. Iris bright orange-gold with greenish tint in lower half and fine black reticulations throughout. Iris periphery lined with black. Sclera light yellowish-green.

**Coloration in preservative:** In preservative, coloration of holotype significantly faded to light brown on dorsum and flanks, with slightly paler limbs and beige on ventral sides (Figure 3B); dark markings on dorsal surfaces brownish, dark banding on dorsal surface of tibiotarsus, antebrachium, hands, and feet well-discernable (Figure 3A). Elbows and upper arms pale brown. White speckles on ventral surface not discernable. Macroglands creamy white.

**Measurements of holotype (in mm):** SVL 28.0; HW 9.9; HL 10.7; ED 4.3; TyD 1.9; E-T 0.8; E-S 3.7; E-N 2.0; N-S 2.1; IO 2.9; IN 2.3; ELW 2.7; FAL 7.1; HAL 6.2; FIL 1.8; FIIL 2.6; FIIIL 4.5; FIVL 1.8; TbL 12.5; FeL 11.9; FOL 11.2; TFOL 17.0; IMT 1.8; IFE 5.0; IBE 8.5; TIL 1.1; TIIL 3.3; TIIIL 4.2; TIVL 6.3; TVL 3.0.

**Variation:** All individuals in the type series were generally similar in morphology and body proportions; measurements of the type series are shown in Table 3 and representative photograph of male paratype in life is shown in Figure 5. Eyes were notably protuberant in living specimens (Figure 5). All specimens showed certain variation in darker brown patterns on dorsum and dark bands on shanks, forearms, hands, and feet. The single known female (PYU DTD-509) was slightly larger (SVL 30.3 mm) than the holotype and two paratype males. Skin texture appeared to be much less tuberculate in preservative (Figure 3) than in life (Figure 5).

**Comparisons:** *Leptolalax macrops* **sp. nov.** differs from all other *Leptolalax* species in mainland Southeast Asia based on morphology.

*Leptolalax macrops* **sp. nov.** can be distinguished from all congeners that are not members of the *L. applebyi* species group in its overall morphology. *Leptolalax macrops* **sp. nov.** can be differentiated from all *Leptolalax* species south of the Isthmus of Kra currently assigned in the subgenus *Leptolalax* (*L. arayai*, *L. dringi*, *L. fritinniens*, *L. gracilis*, *L. hamidi*, *L. heteropus*, *L. kajangensis*, *L. kecil*, *L. marmoratus*, *L. maurus*, *L. pictus*, *L. platycephalus*, *L. sabahmontanus*, and *L. solus*) in having pectoral and ventrolateral macroglands (vs. absent) and supra-axillary glands (vs. absent in most species, except *L. marmoratus*). With its medium body size (SVL 28.0–29.3 mm in adult males, 30.3 mm in single adult female), the new species can be distinguished from larger congeners, including *L. bourreti* (males 28.0–36.2 mm, females 42.0–45.0 mm), *L. eos* (males 33.1–34.7 mm, female 40.7 mm), *L. nahangensis* (male 40.8 mm), *L. platycephalus* (male 35.1 mm, female 46.0 mm), *L. sungi* (males 48.3–52.7 mm, females 56.7–58.9 mm), and *L. zhangyapingi* (males 47.6–50.7 mm); and from smaller-sized species, including *L. alpinus* (males 24.0–26.4 mm), *L. croceus* (males 22.2–27.3 mm), *L. isos* (males 23.7–27.9 mm), *L. kecil* (males 19.3–20.5 mm, female 25 mm), *L. khasiorum* (males 24.5–27.3 mm), *L. laui* (males 24.8–26.7 mm), *L. pluvialis* (males 21.3–22.3 mm), and *L. tengchongensis* (males 23.9–26.0 mm). With its distinct tympanum, *Leptolalax macrops* **sp. nov.** differs from *L. tuberosus*, *L. croceus*, and *L. sungi* (vs. tympanum hidden in the latter species). With its roughly granular dorsum with larger tubercles, *Leptolalax macrops* **sp. nov.** differs from *L. alpinus*, *L. bourreti*, *L. fuliginosus*, *L. gracilis*, *L. hamidi*, *L. heteropus*, *L. isos*, *L. kajangensis*, *L. kalonensis*, *L. liui*, *L. melanoleucus*, *L. minimus*, *L. nahangensis*, *L. oshanensis*, *L. pelodytoides*, *L. pictus*, and *L. pluvialis* (vs. mostly smooth skin with or without skin ridges) and from *L. croceus* and *L. tuberosus* (vs. highly tuberculate dorsum). With its greyish-violet ventral surface with rare white speckling, the new species also differs from *L. croceus* (vs. orange belly); from *L. aereus*, *L. bourreti*, *L. eos*, *L. firthi*, *L. fuliginosus*, *L. isos*, *L. khasiorum*, *L. lateralis*, *L. laui*, *L. liui*, *L. minimus*, *L. nahangensis*, *L. nokrekensis*, *L. nyx*, *L. oshanensis*, *L. pelodytoides*, *L. solus*, *L. sungi*, *L. tamdil*, *L. tuberosus*, and *L. zhangyapingi* (vs. mostly white, creamy white, or pale grey ventral surfaces with or without dark spots or mottling); from *L. alpinus*, *L. maoershanensis*, *L. melanoleucus*, *L. pluvialis*, *L. tengchongensis*, and *L. ventripunctatus* (vs. large patches of distinct brown/grey and white marbling or blotches); from *L. petrops* (vs. pale pink and slightly translucent belly, ventral surface of chest and abdomen immaculate white); and from *L. kecil* (vs. uniformly dark venter with large, dark orange pectoral glands). The new species can be further distinguished from *L. aereus*, *L. croceus*, *L. eos*, *L. firthi*, *L. isos*, *L. laui*, and *L. tuberosus* by having a supratympanic fold with a distinct dark brown to black line (vs. dark supratympanic line absent in the latter species). With its toes showing basal webbing and no lateral fringing, *Leptolalax macrops* **sp. nov.** can be diagnosed from *L. aereus*, *L. eos*, *L. firthi*, *L. isos*, *L. khasiorum* and *L. tamdil* (vs. extensive toe webbing and distinct lateral fringes on toes). The new species can be further differentiated from *L. botsfordi* (Lao Cai Province, northern Vietnam) by having a bicolored golden green iris (vs. uniformly brownish-golden iris), greyish-violet ventral coloration (vs. reddish-brown belly with white speckling), and roughly granulate dorsum (vs. weakly shagreened dorsum in *L. botsfordi*). *Leptolalax macrops* **sp. nov.** can be further diagnosed from *L. puhoatensis* (Nghe An Province, northern Vietnam) by its larger size in males (SVL 28.0–29.3 vs. SVL 24.2–28.1 mm), roughly granulate dorsum, not forming dermal ridges in life (vs. distinct dermal ridges present), and tympanum lighter than supratympanic fold (vs. tympanum completely dark).

*Leptolalax macrops* **sp. nov.** is most similar to members of the *L. applebyi* species group inhabiting the Central Highlands of central and southern Vietnam and the northeastern part of Cambodia, including *L. applebyi*, *L. ardens*, *L. bidoupensis*, *L. kalonensis*, *L. maculosus*, *L. melicus*, *L. pallidus*, *L. pyrrhops*, and *L. tadungensis*. Superficially, the new species resembles *L. pyrrhops*, another medium-sized member of the *L. applebyi* species group with large eyes, distributed in the western part of Langbian Plateau (Lam Dong Province) (Poyarkov et al., 2015a). Comparisons of the new species with members of the *L. applebyi* species group are thus appropriate.

From other members of the *L. applebyi* species group, *Leptolalax macrops* **sp. nov.** can be distinguished by a combination of morphological characters (Rowley et al., 2016; the following morphometric differences refer to males only). In body size, *Leptolalax macrops* **sp. nov.** (SVL 28.0–29.3 mm, mean 28.6 mm, *n*=3) differs from all other members of the *L. applebyi* species group, except for *L. kalonensis*, including smaller species *L. applebyi*, *L. ardens*, *L. bidoupensis*, *L. maculosus*, *L. melicus*, *L. pallidus*, and *L. tadungensis* (vs. SVL 19.6–22.3 mm, mean 20.8 mm, *n*=9, in *L. applebyi*; SVL 21.3–24.7 mm, mean 22.8 mm, *n*=16, in *L. ardens*; SVL 18.5–25.4 mm, mean 23.6 mm, *n*=12, in *L. bidoupensis*; SVL 24.2–26.6 mm, mean 25.5 mm, *n*=3, in *L. maculosus*; SVL 19.5–22.7 mm, mean 20.7 mm, *n*=8, in *L. melicus*; SVL 24.5–27.7 mm, mean 25.6 mm, *n*=8, in *L. pallidus*; SVL 23.3–28.2 mm, mean 25.0 mm, *n*=10, in *L. tadungensis*), and larger species *L. pyrrhops* (vs. SVL 30.8–34.3 mm, mean 33.2 mm, *n*=7, in *L. pyrrhops*). The new species differs from other members of the *L. applebyi* species group, with the exception of *L. pyrrhops*, in having a much larger eye diameter (ED/SVL 0.15–0.16, mean 0.16, *n*=3, in the new species vs. 0.10–0.13, mean 0.12, *n*=9, in *L. applebyi*; vs. 0.12–0.14, mean 0.13, *n*=16, in *L. ardens*; vs. 0.11–0.14, mean 0.12, *n*=12, in *L. bidoupensis*; vs. 0.12–0.15, mean 0.13, *n*=16, in *L. kalonensis*; vs. 0.12–0.14, mean 0.13, *n*=3, in *L. maculosus*; vs. 0.13–0.14, mean 0.13, *n*=8, in *L. melicus*; vs. 0.12–0.14, mean 0.13, *n*=8, in *L. pallidus*; vs. 0.11–0.14, mean 0.13, *n*=10, in *L. tadungensis*). *Leptolalax macrops* **sp. nov.** has a narrower head than that of *L. maculosus* (HW/SVL 0.35–0.36, mean 0.36, *n*=3 vs. 0.37–0.38, mean 0.37, *n*=3) and *L. pyrrhops* (HL/HW 1.03–1.08, mean 1.06, *n*=3 vs. 1.12–1.31, mean 1.14, *n*=7). The new species has a shorter eye-tympanum distance (E-T/SVL 0.03–0.03, mean 0.03, *n*=3) than *L. maculosus* (vs. E-T/SVL 0.04–0.05, mean 0.04, *n*=3), *L. pallidus* (vs. E-T/SVL 0.04–0.06, mean 0.05, *n*=8), and *L. pyrrhops* (vs. E-T/SVL 0.04–0.06, mean 0.05, *n*=7). *Leptolalax macrops* **sp. nov.** has a shorter tibia (TbL/SVL 0.44–0.45, mean 0.45, *n*=3, in the new species) than that of *L. kalonensis* (vs. TbL/SVL 0.45–0.52, mean 0.48, *n*=16), *L. maculosus* (vs. TbL/SVL 0.48–0.50, mean 0.50, *n*=3), *L. pallidus* (vs. TbL/SVL 0.45–0.51, mean 0.49, *n*=8), and *L. pyrrhops* (vs. TbL/SVL 0.48–0.50, mean 0.50, *n*=7).

*Leptolalax macrops* **sp. nov.** is unique among members of the *L. applebyi* species group in having rudimentary webbing between fingers I–II and II–III (vs. finger webbing absent in other species) and in having rudimentary webbing between its toes (vs. toe webbing absent in *L. ardens*, *L. kalonensis*, *L. maculosus*, *L. pallidus*, *and L. tadungensis*). *Leptolalax macrops* **sp. nov.** can be further distinguished from most *L. applebyi* species group members, except for *L. pallidus* and *L. pyrrhops*, in having roughly granulate skin on dorsum with larger tubercles (vs. smooth to weakly shagreened skin in *L. applebyi*, *L. ardens*, *L. bidoupensis*, *L. kalonensis*, *L. maculosus*, *L. melicus*, and *L. tadungensis*); *L. pallidus* has tuberculate skin and *L. pyrrhops* has dorsum varying from finely shagreened to tuberculate. By lacking dermal fringes on its toes, *Leptolalax macrops* **sp. nov.** can be distinguished from *L. bidoupensis*, *L. maculosus*, and *L. tadungensis* (vs. weak or distinct lateral fringes on toes). In having creamy white supra-axillary glands, the new species differs from other species of the *L. applebyi* species group, except for *L. applebyi* and *L. bidoupensis*, all of which have copper to orange supra-axillary glands. The new species can be further distinguished from *L. pallidus* by having a distinct black supratympanic line (vs. black supratympanic line absent). With its golden/greenish bicolored iris, *Leptolalax macrops* **sp. nov.** can be further distinguished from *L. applebyi*, *L. ardens*, *L. melicus*, and *L. tadungensis* (vs. uniform coloration of iris).

**Distribution:** The new species is currently known from only two sites (~30 km from each other) in the tropical evergreen forests of Phu Yen Province (Hon Den Mt., Ea Ly and Ea Trol Commune border, Song Hinh District) and Dak Lak Province (Chu Mu Mt., M’Drak District, based on molecular data from Rowley et al., 2015a). The new species inhabits the northeastern outcrops of the Langbian Plateau. In Phu Yen Province, *Leptolalax macrops* **sp. nov.** was recorded at elevations between 471 and 630 m a.s.l.. The distribution of the new species may be quite narrow, possibly restricted to a small mountain ridge located on the border of Dak Lak, Khanh Hoa, and Phu Yen provinces.

**Figure 6:**
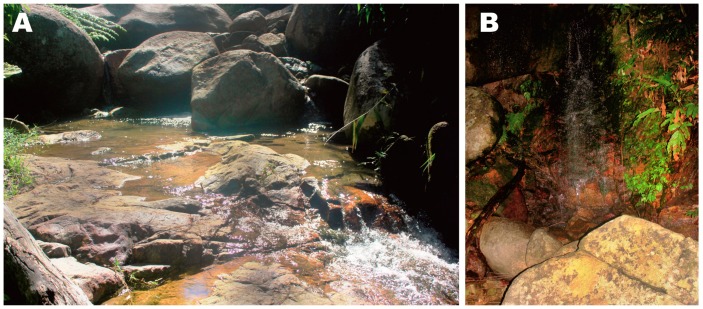
Typical habitat (A) and microhabitat (B) of *Leptolalax macrops* sp. nov. in type locality: Suoi Khi Stream, Hon Den Mt., Ea Ly and Ea Trol commune border, Song Hinh District, Phu Yen Province, Vietnam (Photos by Dang Trong Do)

**Ecological notes:** *Leptolalax macrops* **sp. nov.** is currently known only from evergreen tropical forests on the border of Phu Yen, Dak Lak, and Khanh Hoa provinces. All specimens of the new species were found along cascade rocky streams at elevations between 471–630 m a.s.l. (Figure 6). Surrounding habitat was evergreen tropical forest of large and medium hardwoods and shrubs, with varying degrees of disturbance. Animals were collected at night between 1900–2300 h. Both males and females were found on rocks mid-stream and up to 1 m from the streams.

Frogs were recorded along a cascading stream.

*Leptolalax macrops* **sp. nov.** is known to occur in syntopy with *Microhyla berdmorei* (Blyth) (Do et al., 2017b). Other anuran species recorded in Song Hinh District include *Ophryophryne* cf. *synoria* Stuart, Sok & Neang (indicated as *O. hansi* Ohler by Do et al., 2017b, identification following Poyarkov et al., 2017); *Calluella guttulata* (Blyth); *Kalophrynus* cf. *honbaensis* Vassilieva, Galoyan, Gogoleva & Poyarkov (Vassilieva et al., 2014); *Kaloula indochinensis* Chan, Blackburn, Murphy, Stuart, Emmett, Ho & Brown; *Microhyla mukhlesuri* Hasan, Islam, Kuramoto, Kurabayashi & Sumida (identification following Poyarkov et al., 2014, Yuan et al., 2016); *Microhyla pulchra* (Hallowell), *Fejervaria limnocharis* (Gravenhorst); *Limnonectes* cf. *bannaensis* Ye, Fei & Jiang; *Limnonectes poilani* (Bourret); *Occidozyga lima* (Gravenhorst); *Sylvirana nigrovittata* (Blyth); *Odorrana* cf. *morafkai* (Bain, Lathrop, Murphy, Orlov & Ho); *Polypedates mutus* (Smith), and *Rhacophorus annamensis* Smith (data from Do et al., 2015, 2017b).

**Conservation status:** To date, the new species is only known from a small montane area on the border of Dak Lak, Khanh Hoa, and Phu Yen provinces. It is likely that the range of *Leptolalax macrops* **sp. nov.** is quite narrow. The species probably inhabits Ea So Nature Reserve (Dak Lak Province); however, additional research in this area is needed. The new species appears to require closed evergreen forest along the streams where it occurs. Areas of low to middle elevation montane tropical forest are greatly endangered in the southern coastal areas of Vietnam, including Phu Yen Province. Given the available information, we suggest the species should be considered as Data Deficient following the IUCN’s Red List categories (IUCN, 2001) until the distribution and habitat requirements of the new species are more fully documented.

## DISCUSSION

Our molecular data revealed hidden diversity of the *L. applebyi* species group, with additional herpetological surveys in mountain areas of Indochina possibly leading to the discovery of further new lineages and species of *Leptolalax*. Our finding brings the number of recognized species of the genus *Leptolalax* to 54, and the number of *Leptolalax* species known from Vietnam to 24.

The herpetofauna of the Phu Yen Province is poorly studied compared to the adjacent provinces of Dak Lak and Khanh Hoa. Nguyen et al. (2009) recorded 12 species of reptiles and only five species of amphibians from this province. Ziegler et al. (2013) described a new species of bent-toed gecko, *Cyrtodactylus kingsadai* Ziegler, Phung, Le & Nguyen, from Tuy Hoa District in the eastern part of Phu Yen Province. Recently, Do et al. (2017a) reported *Lycodon cardamomensis* Daltry & Wüster for the first time from Vietnam based on a single specimen collected from Phu Yen Province. More recently, Do et al. (2017b) reviewed the available data on amphibian species found in Phu Yen Province, and added eight new provincial records and listed 33 species of amphibians for the province. The present paper describes a new species of *Leptolalax* from the Song Hinh District. We also recorded a previously undescribed lineage of *Leptolalax* sp. from Tay Hoa District of Phu Yen Province; however, further morphological and molecular research is required to clarify the taxonomic status of this population.

Tropical forests are greatly endangered throughout Southeast Asia, including Vietnam. Compared with the hard-to-access montane tropical forests in the Annamite Mountains, evergreen tropical forests in lowland and foothill areas of the southern coastal region of Vietnam are more endangered; most areas of lowland tropical forest are already destroyed due to logging and other human activities (De Koninck, 1999; Laurance, 2007; Meijer, 1973; Meyfroidt & Lambin, 2008). However, despite their accessibility, the many remaining patches of tropical forest could cradle still unknown biodiversity, which makes the need for biological exploration in this region even more urgent.

## ADDENDUM

During the revision process of the present manuscript, a new paper by Nguyen et al. (2018) was published providing description of *Leptolalax rowleyae*: Nguyen, Poyarkov, Le, Vo, Phan, Duong, Murphy & Nguyen, 2018, a new species of the *L. applebyi* group from the Son Tra Peninsula in Da Nang City, central Vietnam (published on 1 March 2018). We were unable to include *L. rowleyae* in the comparisons section or phylogenetic analysis in the present manuscript; however, it is markedly distinct from the new species in a number of morphological attributes: by smaller body size: SVL 23.4–25.4 mm in males and 27–27.8 mm in females (vs. SVL 28.0–29.3 mm in adult males and 30.3 mm in single adult female of the new species); by pinkish milk-white to light brown ventral surface with numerous white speckles (vs. greyish-violet ventral surface with rare white speckling in the new species); and by much smaller eyes, ED/SVL 0.08–0.11 in males (vs. ED/SVL 0.15–0.16 in males of the new species) (data from Nguyen et al., 2018). The new species can also be distinguished from *L. rowleyae* by deep divergence in the 16S rRNA mtDNA gene (*P*-distance 12.60%) and phylogenetic position (the new species is mentioned as “*Leptolalax* sp.” in the work of Nguyen et al., 2018: Figure 1).

In addition, a recently accepted manuscript by Chen et al. (2018) (published online on 10 March 2018) provides a novel multi-locus phylogenetic hypothesis for the genus *Leptolalax*, describing the latter as a synonym of the genus *Leptobrachella* Smith, 1925. Due to the simultaneous review period of the present paper and the work of Chen et al. (2018), we were unable to implement the new taxonomy at the stage of submission and reviewing process. We suggest that the new species *Leptolalax macrops* **sp. nov.** should hereafter be referred to as *Leptobrachella macrops* Duong, Do, Ngo, Nguyen & Poyarkov to reflect the revised taxonomy.

## APPENDIX I

Examined material, museum IDs given in bold.

Abbreviations: VNMN: Vietnam National Museum of Nature, Hanoi (Vietnam); ZMMU: Zoological Museum of Lomonosov Moscow State University, Moscow (Russia); ZISP: Zoological Institute R.A.S., St. Petersburg (Russia).

*Leptolalax aereus*: **ZISP 12042–12045** (Vietnam, Quang Binh Pr., Phong Nha – Ke Bang N.P.; 4 sp.); **ZMMU A-5214** (Vietnam, Quang Binh Pr., Phong Nha – Ke Bang N.P.; 2 sp.).

*Leptolalax applebyi*: **ZMMU A-5529** (Vietnam, Thua Thien - Hue Pr., A Roang area; 15 sp.); **ZMMU A-5556** (Vietnam, Thua Thien - Hue Pr., A Roang area; 1 sp.).

*Leptolalax ardens*: **ZMMU NAP-06099–06100** (Vietnam, Gia Lai Pr., Kon Ka Kinh N.P., 2 sp.).

*Leptolalax bidoupensis*: **ZMMU A-4717** (Vietnam, Lam Dong Pr., Bidoup – Nui Ba N.P., Bidoup Mt.; 1 sp.); **ZMMU A-4797** (Vietnam, Lam Dong Pr., Bidoup – Nui Ba N.P., Bidoup Mt., Hon Giao Mt.; 4 sp.); **ZMMU A-5211** (Vietnam, Lam Dong Pr., Bidoup – Nui Ba N.P., Bidoup Mt.; 1 sp.).

*Leptolalax bourreti*: **ZISP 12046**, **ZISP 12048** (Vietnam, Lao Cai Pr., Sa Pa, Hoang Lien N.P.; 2 sp.); **ZMMU A-5220** (Vietnam, Lao Cai Pr., Sa Pa, Hoang Lien N.P.; 1 sp.); **ZISP 12048–12050** (Vietnam, Lao Cai Pr., Van Ban N.R.; 3 sp.); **ZMMU A-5219** (Vietnam, Lao Cai Pr., Van Ban N.R.; 1 sp.); **ZMMU A-5031** (Vietnam, Lao Cai Pr., Sa Pa, Tram Don, Fansipan Mt., Hoang Lien N.P.; 6 sp.).

*Leptolalax firthi*: **ZISP 12091**, **ZISP 12058**, **ZISP 12051–12057**, **ZISP 12092–12093** (Vietnam, Kon Tum, Ngoc Linh N.P., Dac Glei; 11 sp.); **ZMMU A-5210** (Vietnam, Kon Tum, Ngoc Linh N.P., Dac Glei; 5 sp.).

*Leptolalax heteropus*: **ZMMU NAP-06788** (Malaysia, Perak, Larut hills; 1 sp.).

*Leptolalax nahangensis*: **ZMMU VNH10, ZMMU VNH16** (Vietnam, Bak Kan Pr., Na Hang; 2 sp.).

*Leptolalax nyx*: **ZISP12059–12061** (Vietnam, Ha Giang Pr., Ha Giang; 3 sp.).

*Leptolalax pallidus*: **ZMMU NAP-01740** (Vietnam, Lam Dong Pr., Bidoup – Nui Ba N.P., Giang Ly St.; 1 sp.); **ZMMU ABV-00453** (Vietnam, Lam Dong Pr., Bidoup – Nui Ba N.P., Bidoup Mt.; 1 sp.).

*Leptolalax petrops*: **ZMMU NAP-06537**; **ZMMU NAP-06565**; **ZMMU NAP-06567** (Vietnam, Phu Tho Pr., Xuan Son N.P.).

*Leptolalax pluvialis*: **ZISP 12075–12081** (Vietnam, Lao Cai Pr., Sa Pa, Hoang Lien N.P., Tram Don; 7 sp.); **ZMMU A-5209** (Vietnam, Lao Cai Pr., Sa Pa, Hoang Lien N.P., Tram Don; 4 sp.); **ZMMU A-5222** (Vietnam, Lao Cai Pr., Sa Pa, Tram Don, Fansipan Mt.; 8 sp.).

*Leptolalax pyrrhops*: **ZMMU A-5208** (Vietnam, Loc Bac Forest Enterprise, Loc Bao Comm., Bao Lam Distr., Lam Dong Pr., Vietnam; holotype); **ZMMU A-4873** (Vietnam, Loc Bac Forest Enterprise, Loc Bao Comm., Bao Lam Distr., Lam Dong Pr., Vietnam; 6 sp., paratypes); **ZISP 12041** (Vietnam, Loc Bac Forest Enterprise, Loc Bao Comm., Bao Lam Distr., Lam Dong Pr., Vietnam; paratype); **VNMN A2015.02** (Vietnam, Loc Bac Forest Enterprise, Loc Bao Comm., Bao Lam Distr., Lam Dong Pr., Vietnam; paratype).

*Leptolalax sungi*: **ZMMU A-4349** (Vietnam, Ha Giang Pr., Ha Giang; 3 sp.).

*Leptolalax tuberosus*: **ZMMU A-4110** (Vietnam, Kon Tum Pr., Kon Plong; 1 sp.); **ZISP 12094–12095** (Vietnam, Kon Tum Pr., Kon Plong; 2 sp.); **ZMMU A-5213** (Vietnam, Kon Tum Pr., Kon Plong; 1 sp.).

*Leptolalax ventripunctatus*: **ZMMU A-5223** (Vietnam, Dien Bien Pr., Muong Nhe N.R., Sin Hau St.; 12 sp.); **ZMMU A-5156** (Vietnam, Dien Bien Pr., Muong Nhe N.R., Sin Hau St.; 5 sp.); **ZMMU A-5225** (Vietnam, Phu Tho, Xuan Son N.P.; 3 sp.); **ZMMU A-5224** (Vietnam, Phu Tho, Xuan Son N.P.; 1 sp.); **ZISP 12062–12074** (Vietnam, Vinh Phuc Pr., Tam Dao N.P.; 13 sp.); **ZMMU A-5212** (Vietnam, Vinh Phuc Pr., Tam Dao N.P.; 5 sp.).
